# Supplementary material for: La(OH)3 Multi-Walled Carbon Nanotube/Carbon Paste-Based Sensing Approach for the Detection of Uric Acid—A Product of Environmentally Stressed Cells
Source: Biosensors (Basel). 2022 Sep 1;12(9):705. doi: 10.3390/bios12090705 (PMC9496040; doi:10.3390/bios12090705)
Supplement: Supplementary file 1 [file biosensors-12-00705-s001.zip › biosensors-1857865-supplementary.pdf]

biosensors

MDPI

Supplementary

La(OH)3 Multi-Walled Carbon Nanotube/Carbon Paste-Based Sensing Approach for the Detection of Uric Acid—A Product of Environmentally Stressed Cells

Sara Knežević [1,2], Miloš Ognjanović [3], Vesna Stanković [4], Milena Zlatanova [1], Andrijana Nešić [1], Marija Gavrović-Jankulović [1] and Dalibor Stanković [1,5,*]

[1] Faculty of Chemistry, University of Belgrade, Studentski trg 12-16, 11000 Belgrade, Serbia;
[2] University of Bordeaux, CNRS, Bordeaux INP, Institut des Sciences Moléculaires, UMR 5255, 33400 Talence, France
[3] Department of Theoretical Physics and Condensed Matter Physics, "VINČA" Institute of Nuclear Sciences-National Institute of the Republic of Serbia, University of Belgrade, 11000 Belgrade, Serbia
[4] Scientific Institution, Institute of Chemistry, Technology and Metallurgy, National Institute University of Belgrade, 11000 Belgrade, Serbia
[5] Department of Radioisotopes, "VINČA" Institute of Nuclear Sciences-National Institute of the Republic of Serbia, University of Belgrade, 11000 Belgrade, Serbia
* Correspondence: dalibors@chem.bg.ac.rs

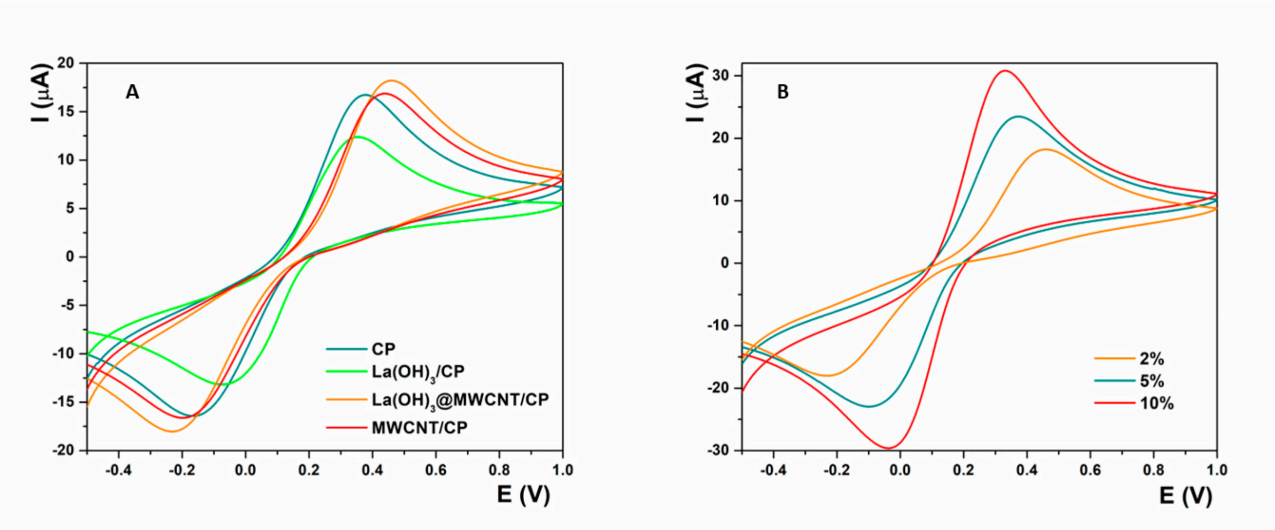

Figure S1. Cyclic voltammograms in 5mM $Fe^{2+/3+}$ in 0.1 M KCl and 0.1 M PB pH 6, in the potential range from −0.5 V to 1 V at the 50mV/s scan rate of **A)** unmodified CP electrode and electrodes modified with 2% of $La(OH)_3$, MWCNT and $La(OH)_3$@MWCNT **B)** electrodes modified with 2%, 5% and 10% of $La(OH)_3$@MWCNT.

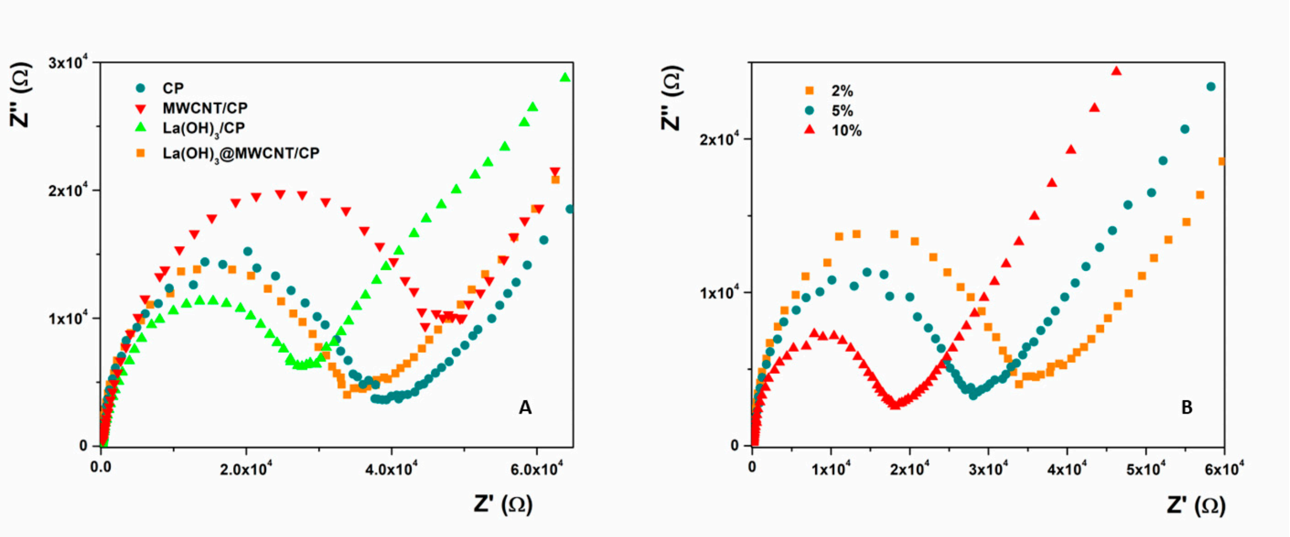

Figure S2. EIS spectra in 5 mM $Fe^{2+/3+}$ in 0.1M KCl and 0.1 M PB pH 6 on 0.3 V and in the frequency range from 10 kHz to 10 mHz of **A)** unmodified CP electrode and electrodes modified with 2% of $La(OH)_3$, MWCNT and $La(OH)_3$@MWCNT **B)** electrodes modified with 2%, 5% and 10% of $La(OH)_3$@MWCNT.

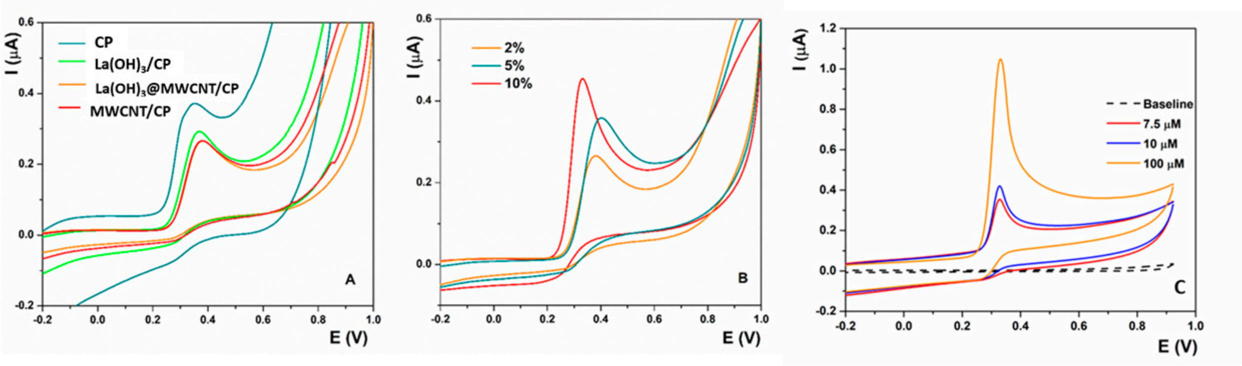

Figure S3. (A) CV in 0.1 M PB pH 6 in the presence of 10 µM UA - unmodified CP electrode and electrodes modified with 2% of $La(OH)_3$, MWCNT and $La(OH)_3$@MWCNT **(B)** CV in 0.1 M PB pH 6 in the presence of 10 µM UA - electrodes modified with 2%, 5% and 10% of $La(OH)_3$@MWCNT **(C)** CV in 0.1 M PB pH 6 in the presence of 7.5; 10 and 100 µM UA using electrode modified with 10% of $La(OH)_3$@MWCNT.

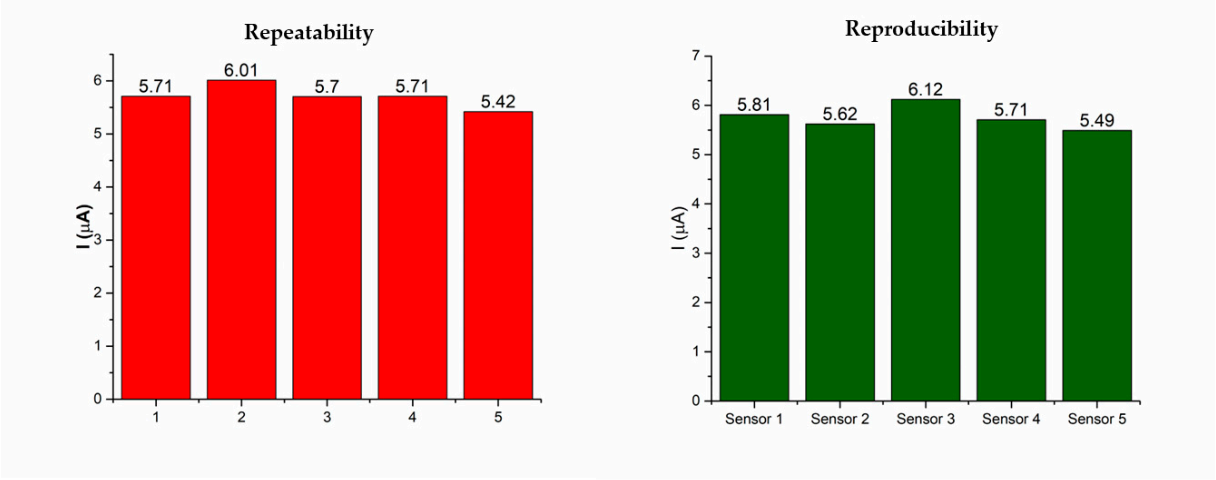

Figure S4. Repeatability and reproducibility studies for proposed sensor.

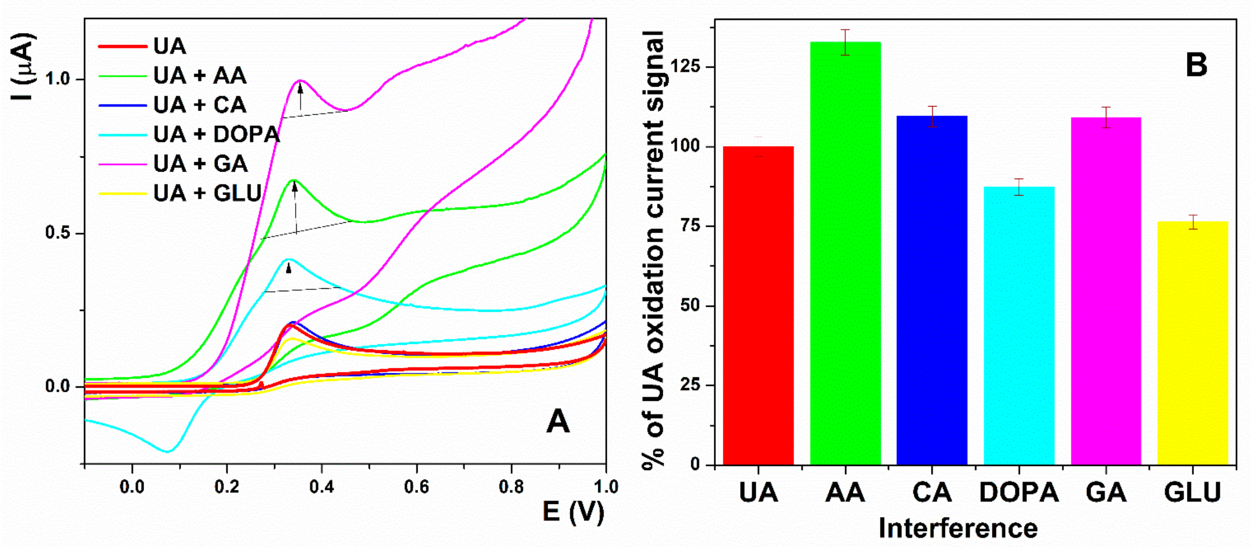

Figure S5. Studying the impact of potential interferents on UA detection **A)** CV in 0.1 M PB pH 6, at the potential range from −0.05 V to 1 V at the scan rate 50 mV/s, **B)** Histogram – percent of the anodic peak current which arose from the oxidation of UA.

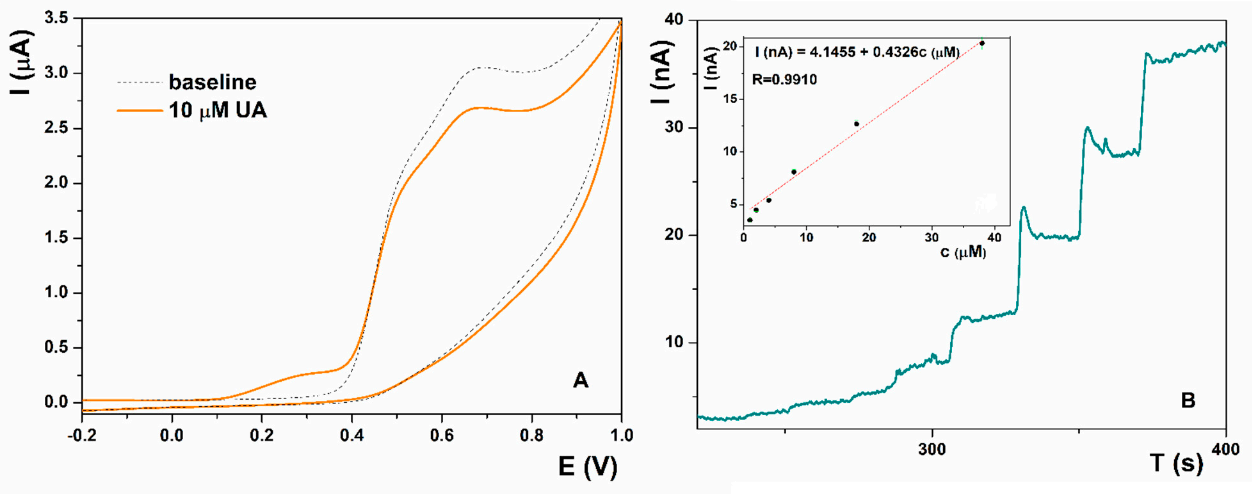

Figure S6. (A) CV in the potential range from 0.5 V to 1 V at the scan rate of 50 mV/s in the biological matrix in the presence and absence of UA **(B)** Amperometric curve of UA in the real sample matrix **(inset B)** Calibration curve of UA standard solutions in the biological matrix.
